# Arecoline as a Novel Scaffold Targeting the ATAD2 Bromodomain for Cell Cycle Modulation

**DOI:** 10.3390/pharmaceutics18030324

**Published:** 2026-03-03

**Authors:** Ting-Syuan Lin, Jingting Wan, Jingjin He, Shidong Cui, Yun Huang, Bojian Zhang, Hsi-Yuan Huang, Kexin Zhu, Jihang Chen, Tao Zhang, Shangfu Li, Liao Hu, Yongfei Wang, Hsien-Da Huang, Ping Tang, Yang-Chi-Dung Lin

**Affiliations:** 1 Warshel Institute for Computational Biology, School of Medicine, The Chinese University of Hong Kong, Shenzhen 518172, China; tingsyuanlin@link.cuhk.edu.cn (T.-S.L.); jingtingwan@link.cuhk.edu.cn (J.W.); shidongcui@link.cuhk.edu.cn (S.C.); bojianzhang@link.cuhk.edu.cn (B.Z.); huanghsiyuan@cuhk.edu.cn (H.-Y.H.); kexinzhu1@link.cuhk.edu.cn (K.Z.); chenjihang@cuhk.edu.cn (J.C.); lishangfu@cuhk.edu.cn (S.L.); yfwang@cuhk.edu.cn (Y.W.); 2 Guangdong Provincial Key Laboratory of Digital Biology and Drug Development, The Chinese University of Hong Kong, Shenzhen 518172, China; 3 Department of General Practice, Shenzhen Luohu People’s Hospital, Shenzhen 518000, China; jingjin@hotmail.com (J.H.); yhuangbr@connect.ust.hk (Y.H.); 4 Luohu Clinical College, Shantou University Medical College, Shenzhen 518000, China; 5 Better Way Group—Chinese University of Hong Kong (Shenzhen) Warshel Joint Laboratory for Skin Health and Active Molecule Innovation, Shenzhen 518172, China; zhangt@mistinechina.com; 6 Department of Electrical & Computer Engineering, University of Illinois at Chicago, Chicago, IL 60607, USA; lhu231@my.trine.edu; 7 Department of Endocrinology, Key Laboratory of Endocrinology of National Ministry of Health, Peking Union Medical College Hospital, Chinese Academy of Medical Sciences & Peking Union Medical College, Beijing 100730, China

**Keywords:** arecoline, target identification, ATAD2, cell cycle, lead optimization

## Abstract

**Background/Objectives**: ATPase family AAA domain-containing protein 2 (ATAD2) is an oncogenic chromatin regulator that amplifies E2F/MYC transcriptional programs, yet direct modulators remain scarce. Arecoline (ARE), the primary alkaloid of the areca nut, is a known carcinogen but paradoxically exhibits context-dependent anti-proliferative activities. In this study, we resolve this paradox by defining ARE’s anti-cancer mechanism. **Methods**: Breast cancer cell proliferation and colony formation assays were performed to evaluate the anti-proliferative effects of ARE. Cell-cycle distribution was analyzed to determine phase-specific effects. Transcriptomic profiling was conducted to identify affected gene networks. An unbiased Cellular Thermal Shift Assay–Mass Spectrometry (CETSA-MS) screening was used to identify direct protein targets, followed by CETSA–Western blotting for validation. Finally, in silico structure-based design was applied to generate novel derivatives with improved predicted properties. **Results**: ARE suppressed breast cancer cell proliferation and colony formation by inducing G1/S phase arrest. Transcriptomic analysis revealed that this phenotype was driven by profound suppression of the E2F/Cell Cycle gene network. CETSA-MS identified ATAD2 through multi-omics convergence, as the 67 direct targets were collectively most significantly enriched in the E2F pathway. CETSA–Western blotting confirmed that ARE binds and thermally stabilizes ATAD2. Mechanistically, ARE engagement of ATAD2 led to downregulation of key downstream proteins, including MYC and Cyclin D1, directly linking target modulation to G1/S arrest. Structure-based design further yielded novel derivatives with predicted enhanced ATAD2 binding and substantially reduced toxicity. **Conclusions**: Together, these findings uncover ATAD2 as a druggable target of ARE, establish proof-of-concept for repurposing this scaffold, and provide a rational framework for developing safer ATAD2-targeted therapies.

## 1. Introduction

ATPase family AAA domain-containing protein 2 (ATAD2) integrates chromatin remodeling with transcriptional regulation. It functions as a transcriptional co-activator for E2Fs and MYC, playing a pivotal role in amplifying transcriptional programs that promote cell cycle progression and cellular proliferation Ciró, Prosperini [1,2]. Consistently, ATAD2 exhibits higher expression across diverse cancer types, with its upregulation strongly linked to increased proliferative capacity and aberrant cellular phenotypes [3,4]. ATAD2 is also identified as an antigen that can trigger immune responses, indicating its dual significance in cellular regulation [5]. Despite its importance in fundamental cellular regulation, specific small-molecule modulators of ATAD2 remain limited, making it a compelling yet underexplored target for mechanism-based research. Notably, its bromodomain has often been characterized as “difficult to drug” due to a shallow and highly polar KAc-binding pocket, together with the pronounced flexibility of the ZA and BC loops, which collectively prevent stable and selective ligand binding [6]. Cell cycle dysregulation is a hallmark of many diseases, where abnormal transcription factors and chromatin remodelers drive excessive cell growth and survival [7]. Evidence implicates ATAD2 as a central regulator of these pathways, highlighting it as a promising target for research into cell cycle modulation.

**Figure 1:**
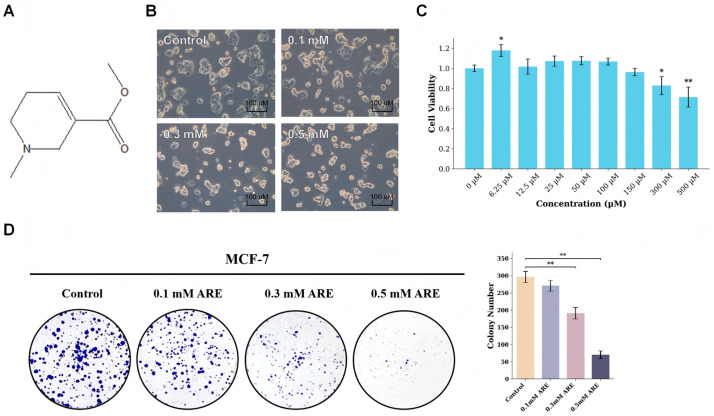
ARE suppresses MCF-7 cell proliferation and viability. (**A**) The structure of Arecoline. (**B**) Microscopic images showed that ARE treatment led to a decrease in cell proliferation. (**C**) Concentration–response analysis of ARE on MCF-7 cell viability, assessed by CCK-8 assay after 12 h of treatment. (**D**) Representative images (**Left**) and quantification (**right**) of the colony formation assay. Cells were treated with the indicated concentrations of ARE for 14 days. Data in (**C**,**D**) are presented as mean ± SD (*n* = 3). Statistical significance was determined by one-way ANOVA followed by Dunnett’s multiple comparisons test. *, *p* < 0.05 vs. CTRL; **, *p* < 0.01 vs. CTRL.

Natural products have historically served as a rich source of structurally diverse scaffolds that can modulate essential cellular processes. Arecoline (ARE), the key alkaloid of the areca nut (*Areca catechu*), exemplifies this chemical complexity (Figure 1). Experimental studies have shown that ARE induces G2/M cell cycle arrest, DNA damage responses, and altered expression of p53 and IL-6 in epithelial and fibroblast cells [8,9,10]. Accordingly, ARE has been widely regarded as a compound associated with adverse health outcomes and oral carcinogenesis [11].

However, in contrast to these well-documented effects, several studies reported that ARE exhibits context- and dose-dependent biological activities. In selected cell lines, ARE has been shown to suppress proliferation, induce G1/S or G2/M phase arrest, and trigger apoptosis, notably at concentrations ranging from approximately 0.1 to 0.8 mM [12,13]. Aligning with these established effective doses for mechanistic investigation, the present study utilizes a concentration range of 0.1–0.5 mM to specifically characterize the molecular targets driving these anti-proliferative phenotypes. These observations suggest that the biological consequences of ARE exposure may vary depending on tissue type, genetic background, and concentration. Thus, while ARE in its natural form poses health risks under chronic exposure, its chemical scaffold may still provide a basis for designing safer analogs targeting key cellular regulators such as ATAD2.

In this study, we first characterize the anti-proliferative effects of ARE, demonstrating its potent induction of G1/S phase arrest. Through transcriptomic analysis, we establish that this phenotype is driven by the profound suppression of the E2F/Cell Cycle gene expression network. To identify the upstream driver of this effect, we employed an unbiased Cellular Thermal Shift Assay–Mass Spectrometry (CETSA-MS) screening and established a powerful multi-omics convergence: the 67 direct protein targets identified by CETSA-MS were, as a group, most significantly enriched for the exact same “E2F targets” pathway. This data-driven approach led us to identify ATAD2, a known master co-activator of E2F, as the primary molecular target. We validate this direct interaction via CETSA-Western Blot and confirm that ARE-mediated ATAD2 targeting suppresses key downstream proteins, including Cyclin D1 and MYC, mechanistically linking the target to the phenotype. Finally, based on this new mechanism, we computationally optimize the ARE scaffold to design novel derivatives with predicted improvements in both ATAD2 binding affinity and safety profiles. This work redefines the mechanism of action for Arecoline and establishes a rational framework for developing structure-based modulators for the challenging ATAD2 bromodomain.

## 2. Materials and Methods

### 2.1. Cell Culture

MCF-7 cells were obtained from Cellcook Biotech Co., Ltd. (Guangzhou, China). The cell line identity was validated by the supplier using Short Tandem Repeat (STR) profiling, and the cells were confirmed to be negative for mycoplasma contamination. Cells were maintained in MEM medium (Gibco, Billings, MT, USA, No. 31095029) supplemented with 10% FBS (Gibco, No. 26170043), 1 × Non-Essential Amino Acids solution (Gibco, Billings, MT, USA, No. 11140050), and 10 μg/mL insulin (Nanjing Duly Biotech Co., Ltd., Nanjing, China, CAS: 11061-68-0). The cells were cultured at 37 °C in a humidified incubator with 5% CO_2_. For cell detachment and subculture, 0.25% trypsin-EDTA was used.

### 2.2. Cell Counting Kit-8 Assay

To evaluate the effective concentration range of ARE (Aladdin Biochemical Technology, Shanghai, China, No. A348021) on MCF-7 cells, cell viability was assessed using the Cell Counting Kit-8 (CCK-8) assay. MCF-7 cells (2.0 × 10^4^ cells/well) were plated in triplicate in a 96-well plate. After 24 h of incubation, the medium was replaced with either fresh medium containing DMSO at the same final concentration used in the treatment groups (control group) or varying concentrations of ARE solution prepared in DMSO (treatment groups) ranging from 0 to 0.5 mM for 12 h. After an additional 12 h incubation, 100 μL of CCK-8 solution (Beyotime, Shanghai, China No. C0038) diluted 1:10 was added to each well and incubated for 2 h at 37 °C. The absorbance was then measured at 450 nm using a microplate reader (BioTek Epoch 2, Santa Clara, CA, USA).

### 2.3. Colony Formation Assay

To assess the effect of ARE on cell proliferation, a colony formation assay was performed. Cell suspensions were diluted in gradient multiples and plated in 6-well plates at densities of 300 cells each well. Three biological replicates were used for each treatment group. After 24 h of incubation, the cells were treated with ARE at concentrations of 0.5, 0.3, 0.1, and 0 mM. The plates were cultured until sufficiently large colonies formed in the control wells, typically after 14 days. Following incubation, the colonies were fixed and stained with crystal violet for analysis.

### 2.4. Transcriptomic and Enrichment Analysis

MCF-7 cells were seeded at a density of 3.34 × 10^6^ cells per 10 cm dish and incubated for 24 h. Subsequently, ARE was administered to the cultures at concentrations of 0.5 mM, 0.3 mM, and 0.1 mM, along with a control group without ARE. Each treatment was performed in triplicate. After 12 h of treatment, cells were collected from each dish using 1 mL of Trizol reagent (Invitrogen, Carlsbad, CA, USA Cat. No. 15596026). Total RNA was isolated from the MCF-7 cells using the Direct-zol™ RNA Miniprep Kit (Irvine, CA, USA No. R2052) according to the manufacturer’s protocol. For subsequent data analysis, paired-end 100 bp reads (PE100) were sequenced on the MGISEQ-2000RS platform (MGI, Shenzhen, China) [14].

Differentially expressed genes (DEGs) were identified from the RNA-seq data using a |fold change|≥2 and an adjusted *p*-value of < 0.05 for statistical significance. The DEGs were then subjected to enrichment analysis using Metascape [15] to identify significantly overrepresented biological processes and pathways.

### 2.5. Cell Cycle Analysis

A cell cycle assay was conducted using a Cell Cycle and Apoptosis Analysis Kit (Beyotime, Shanghai, China C1052). Cells were collected and fixed in 70% ice-cold ethanol at −20 °C overnight. For cell cycle analysis, the cells were centrifuged and resuspended in a solution containing 50 μg/mL propidium iodide (PI) and 20 μg/mL RNase A, followed by incubation at 37 °C for 30 min. Red fluorescence was detected at an excitation wavelength of 488 nm using flow cytometry (CytoFlex, Indianapolis, IN, USA). Data were analyzed using FlowJo software (Tree Star, Inc., Ashland, OR, USA). Debris and doublets were excluded by gating on Forward Scatter (FSC) versus Side Scatter (SSC) and FSC-Height versus FSC-Area, respectively, prior to analyzing the fluorescent signals.

### 2.6. Cellular Thermal Shift Assay–Mass Spectrometry and Western Blotting (CETSA-MS and CETSA-WB)

#### 2.6.1. CETSA-MS

##### Preparation of Cell Lysate for Target Identification

The collected cell pellets were suspended in PBS containing a mixture of 1% protease inhibitors. The cell lysate was extracted by performing three rapid freeze–thaw cycles in liquid nitrogen for lysis. Then, the obtained mixture was centrifuged at 20,000× *g* for 20 min at 4 °C. The supernatant was collected. Its concentration was determined by the BCA method (Coolaber, SK1070-500T, Beijing, China). Before use, it was diluted to 1 mg/mL with lysis buffer.

##### Target Identification of Arecoline

Sample preparation. The sample was prepared by following the method developed by Lyu et al. with some minor modifications [16]. Briefly, the cell lysate was divided into two parts and added either ARE (Aladdin, No. A348021-50mg) dissolved in DMSO (MP, No. 0219605580) or an equal volume of DMSO. The final concentration of ARE was 10 µmol/L. After incubation at room temperature for 10 min, Sera-Mag carboxylate-modified magnetic particles were added at a mass ratio of 5:1. After mixing evenly, the mixture was equally divided into three parallel. The samples were incubated at 52 °C for 3 min and then allowed to stand at room temperature for 3 min. A magnetic rack separated the magnetic beads and the supernatant was discarded. The precipitates were washed twice with PBS. The tris (2-carboxyethyl) phosphine (TCEP) and 2-chloroacetamide (CAA) dissolved in 50 mM N-(2-hydroxyethyl) piperazine-N′-ethanesulfonic acid (HEPES, pH 7.5) buffer were added at a final concentration of 10 and 40 mM, respectively. The samples were heated at 95 °C for 5 min for alkylation. At the end of the reaction, it was cooled at 37 °C and trypsin was added in a 1:25 ratio. The reaction was carried out at 37 °C overnight. The beads were removed with a subsequent magnet, and the supernatant was desalted for proteomic analysis.

LC-MS/MS analysis. The treated peptide samples were subjected to sequential window acquisition of all theoretical mass spectra (SWATH-MS)-based label-free quantitative proteomics analysis using Ekspert nanoLC 400 in tandem with AB Sciex TripleTOF 6600 plus. The peptides were separated by a C18 column (0.3 × 150 mm, 3 µm, 120 Å, AB Sciex). Mobile phase A contained 98% H_2_O with 2% acetonitrile and 0.1% formic acid, while mobile phase B contained 98% acetonitrile with 2% H_2_O and 0.1% formic acid. Gradient elution was performed at a flow rate of 5 µL/min for a total time of 90 min. The gradient table can be found in Table S1. The mass spectrometry experiment parameters were set as follows: CUR: 35; GS1: 18; GS2: 15; ISVF: 5500; TEM: 350; Polarity: Positive; Precursor start mass: 350; Precursor end mass: 1500; Fragment ion start mass: 350; Fragment ion end mass: 1500; MS1 Accumulation Time: 100 ms; MS2 Accumulation Time: 35 ms. The mass range of precursors was divided into 80 windows, and the detailed SWATH dictionary is listed in Table S1.

Data processing. Raw data was processed using DIA-NN 1.9.1 [17]. The DIA mass spectrometry data was analyzed by searching against a spectral library, which was a compendium of highly specific SWATH-MS datasets covering more than 10,000 human proteins [18]. The precursor charge range was set to 2–5. The precursor m/z range was set to 350–1500. The fragment ion *m*/*z* range was set to 100–1500. Match-between-runs (MBR) was enabled. The other parameters were retained as default. The output of a unique protein matrix was used for further analysis. First, a quality control analysis was performed on the data to ensure that there were no abnormal samples. Subsequently, proteins with more than 50% of the missing value were removed. The remaining missing values were interpolated using the median value within the group. After log_2_ transformation, differential expression analyses were performed using Limma to screen differential proteins between drug-treated and vector-treated samples [19].

#### 2.6.2. CETSA-WB

Experiments were conducted to verify the relationship between the compound ARE and its direct target ATAD2. MCF-7 cells were treated with RIPA lysis buffer (Beyotime, Shanghai, China No. P0013C) to extract soluble proteins. Equal amounts of protein were then incubated with ARE (Chengdu Despite Biotechnology Co., Ltd., Chengdu, China Cat No. DH0024-0050) as the treatment group or with DMSO as the negative control group, at room temperature for 10 min prior to the CETSA heat pulse. The samples were aliquoted into PCR tubes and subjected to heating at different temperatures, ranging from 37 °C to 70 °C, using an Applied Biosystems PCR analyzer (Thermo Scientific, Waltham, MA, USA). After heating for 3 min, the samples were cooled at 4 °C for 10 min.

The heated samples were then diluted with 160 μL of DNase-free water, mixed thoroughly, and transferred to centrifuge tubes. The samples were centrifuged at 20,000× *g* for 30 min at 4 °C. The resulting supernatant was mixed with 6 × loading buffer and denatured at 100 °C for 10 min. The denatured samples were immediately transferred to ice and cooled for an additional 10 min. Following cooling, the samples were centrifuged again at 14,000× *g* for 10 min at 4 °C, and the supernatant was collected for Western blot analysis.

### 2.7. Real-Time qPCR

The mRNA expression levels of identified Differentially Expressed Genes (DEGs), including E2F transcription factor 1 (E2F1), E2F transcription factor 2 (E2F2), cyclin D1 (CCND1), cyclin E2 (CCNE2), and MYC proto-oncogene, bHLH transcription factor (MYC), were assessed using qPCR in MCF-7 cell samples. Total RNA was extracted using the Direct-zol™ RNA Miniprep Kit (Irvine, CA, USA Cat. No. R2052), following the manufacturer’s instructions. cDNA was then synthesized using SuperScript III Reverse Transcriptase (Invitrogen, Carlsbad, CA, USA Cat. No. 18080093). The master mix and SYBR Green were combined with total cDNA, and the mixture was loaded into the Applied Biosystems QuantStudio™ 6 Flex Real-Time PCR System for amplification and detection. The primer sequences are provided in Table 1.

### 2.8. Western Blotting

Western blotting was performed according to a previous procedure to confirm differential protein expression corresponding to genes [20]. Using the Genscript eBlot L1 wet transfer system (Genscript, Nanjing, China No. L00686), a comparable amount of protein was separated using 8% SDS-PAGE and then transferred onto polyvinylidene difluoride membranes (Millipore, Burlington, MA, USA). The transplanted membranes were then blocked with QuickBlock™ WesternBlocking Buffer (Beyotime, Shanghai, China Cat. No. P0252) for 15 min at room temperature. Specific primary antibodies, including E2F1 Monoclonal antibody (Proteintech, Rosemont, IL, USA No. 66515-1-Ig), Anti-E2F2 antibody (ABCAM, Cambridgeshire, UK No. ab235837), Cyclin D1 Polyclonal antibody (Proteintech, Rosemont, IL, USA No. 26939-1-AP), Anti-Cyclin E2 antibody (ABCAM, Cambridgeshire, UK No. ab32103), MYC tag Monoclonal antibody (Proteintech, Rosemont, IL, USA No. 60003-2-Ig), and anti-GAPDH (Proteintech, Rosemont, IL, USA No. 60004-1-lg), were then used to probe membranes overnight at 4 °C. The membranes were then supplemented with secondary antibodies that were coupled to horseradish peroxidase (Cell Signaling Technology, Danvers, MA, USA Cat. No. 7076P2/7074P2). After reaction with an electrochemiluminescence (ECL) substrate (Immobilon™ Western, Millipore, Burlington, MA, USA), chemiluminescent signals were detected using an eBlot Touch Imager (eBlot Photoelectric Technology, Shanghai, China). Densitometric analysis was performed using ImageJ software (version 1.49; National Institutes of Health, Bethesda, MD, USA).

### 2.9. Lead Optimization

PocketFlow [21] is a structure-based generative model integrating data-driven learning with explicit chemical constraints. Specifically, the model was pretrained on approximately 8 million drug-like molecules randomly selected from the ZINC database to learn the underlying chemical distribution, and subsequently fine-tuned on a filtered, high-quality subset of CrossDocked2020 (≈150,000 protein–ligand complexes; pose RMSD < 1 Å and ligands with ≤35 heavy atoms after filtering) to capture pocket-conditioned structure–interaction patterns. A customized version of PocketFlow was developed to allow scaffold-guided molecular generation through atom-level constraints, enabling fixation of any user-defined core structure as an initial seed. This customization constrains the generation/sampling process (i.e., enforcing scaffold fixation through atom-level constraints) rather than introducing any additional pretraining or fine-tuning beyond the original PocketFlow training procedure. In this study, ARE was selected as the scaffold. Using this modified model, 1000 candidate molecules targeting the ATAD2 binding pocket were generated to ensure sufficient exploration of chemical space—as evidenced by structural redundancy observed among generated molecules—while maintaining computational feasibility for subsequent docking analysis and property evaluation. To identify promising candidates, an initial filtering was performed by removing molecules with synthetic accessibility (SA) scores greater than 4 [22], quantitative estimate of drug-likeness (QED) scores lower than 0.5 [23], or log*P* values outside the range of 0 to 5 [24]. Molecules that violated more than one of Lipinski’s Rule of Five were also excluded [24]. Subsequently, the remaining compounds were prioritized based on binding affinity and molecular properties. Molecules were first ranked in ascending order according to their AutoDock Vina (version 1.2.5) [25] scores, and those with SA scores not exceeding 3, QED scores above 0.75, and log*P* values between 2 and 3 (allowing slight deviations) were selected, resulting in approximately a dozen candidates. In parallel, molecules were ranked in descending order based on their fm_scores, with only those exhibiting vina scores lower than −8.5 being considered. Applying the same SA, QED, and log*P* criteria, an additional ten molecules were identified. The two sets of selected compounds were merged, and duplicate structures were removed. Further refinement was conducted by ranking the remaining molecules individually according to SA, QED, and log*P* values, and candidates with relatively inferior properties in each category were excluded.

### 2.10. Computational Drug Safety Evaluation

Through the process of lead optimization, 6 optimized lead compounds were obtained. These candidates were subsequently subjected to toxicity and adverse drug reaction (ADR) predictions using ADMETLab platform [26] and DeepADR [27], respectively. ADMETLab is a comprehensive web-based system for physicochemical, pharmacokinetic, and toxicity property prediction, covering endpoints such as hERG inhibition, Ames mutagenicity, acute toxicity, and organ-specific toxicity. DeepADR is a multimodal deep learning framework that integrates chemical structure and drug–target information to estimate the occurrence and frequency of adverse drug reactions, providing calibrated multi-label predictions across 994 ADR categories standardized by MedDRA terminology.

### 2.11. Molecular Docking and Dynamic Simulation

The docking study of the selected target ATAD2 (PDB: 6epj), a ligand for positive control BOH (CID: 134822006) [28] and ARE (CID: 2230) was conducted using CB-dock2 [29]. The crystal structure of the target was obtained from the RCSB protein databank. The ligand structure was obtained from the PubChem database.

After completing the molecular docking simulations, Discovery Studio Visualizer analyzed the docked complexes through 2D interaction diagrams, providing clear insights into the binding interactions. These diagrams illustrated vital features such as hydrogen bonds, hydrophobic interactions, and aromatic contacts. Hydrogen bonds were typically highlighted with dashed lines, showing the bonding direction and strength between the ligand and protein residues. The visualization allowed for identifying critical amino acids involved in binding, enhancing the stability of the ligand–protein complex. Additionally, 3D structures were visualized using the built-in function of CB-Dock2, offering a comprehensive view of the ligand’s orientation and spatial interactions within the active site. This combined 2D and 3D visualization approach simplifies intricate interaction patterns and enables efficient communication and comparison of different ligand binding modes.

To investigate the dynamic stability of prospective bioactive compounds upon target binding, molecular dynamics (MD) simulations were performed using GROMACS 2023.3 [30] computational suite. Small molecule topologies were constructed through the AmberTools24 package with atomic parameterization. The solvation environment for protein-ligand complexes was established employing the TIP3P water model, with ion placement achieved via Monte Carlo methodology [31]. System charge neutralization was attained by adding 0.15 M sodium and chloride ions. All simulations utilized the AMBER99SB force field under periodic boundary conditions. Energy minimization was conducted through a steepest descent algorithm (50,000 steps) with convergence criteria set at 1000 kJ·mol^−1^·nm^−1^ under an NVT ensemble. Subsequent system equilibration employed a V-rescale thermostat to maintain temperature at 310.15 K. Production MD runs were executed in NPT ensemble for 100 ns duration, incorporating Parrinello–Rahman barostat (pressure coupling time constant = 2 ps) and V-rescale thermostat (temperature coupling time constant = 0.5 ps). Covalent bond constraints for hydrogen atoms were maintained using LINCS algorithm. Trajectory analysis included calculation of whole-system root mean square deviation (RMSD) and ligand–protein distance profiles through GROMACS rms module. Intermolecular hydrogen bond formation was quantified using the hbond analysis tool within the simulation package.

## 3. Results

### 3.1. ARE Suppresses Cellular Proliferation and Viability in MCF-7 Cells

The structure of ARE, an alkaloid derived from the areca nut, is shown in Figure 1A. To characterize the primary cellular effects of ARE, we first investigated its impact on MCF-7 cell proliferation and morphology. We observed that ARE treatment significantly altered cellular characteristics. As shown in Figure 1B, treatment for 24 h led to a noticeable decrease in cell number, and morphologically, the cells exhibited changes consistent with reduced proliferative activity.

To quantify this anti-proliferative effect, we performed a cell viability assay at several ARE concentrations. The results indicated that after 12 h of treatment, ARE significantly reduced cell viability in a clear concentration-dependent manner (Figure 1C). Based on the dose–response curve, the estimated half-maximal inhibitory concentration (IC_50_) was calculated to be approximately 0.76 mM. However, at the highest experimental concentration used (0.5 mM), we observed a statistically significant viability reduction of approximately 29% (*p* = 0.028), indicating substantial growth inhibition without complete cytotoxicity. To prioritize mechanistic relevance over non-specific toxicity, we focused on this sub-IC_50_ concentration range (0.1–0.5 mM) for subsequent target identification.

Furthermore, to confirm the impact on long-term proliferative capacity, we conducted a colony formation assay. Quantitative analysis revealed distinct dose-dependent suppression: compared to the control group, ARE treatment reduced colony formation by approximately 36% at 0.3 mM (*p* = 0.001) and by 76% at 0.5 mM (*p* < 0.001) (Figure 1D).

Taken together, these data clearly demonstrate that ARE is a potent inhibitor of cellular proliferation. This key phenotypic observation prompted us to investigate the underlying molecular mechanisms responsible for this anti-proliferative effect, which we explore in the following section.

### 3.2. Transcriptomic Analysis Reveals ARE-Induced G1/S Arrest via Suppression of the E2F/Cell Cycle Pathway

**Figure 2:**
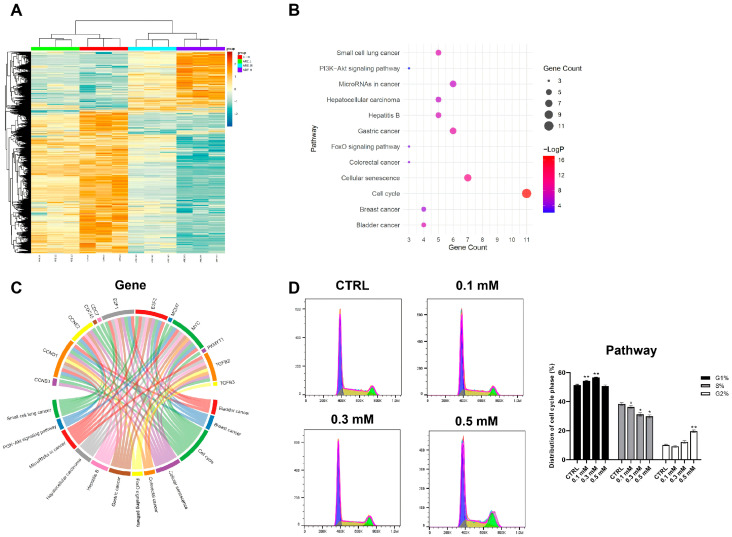
ARE induces G1/S arrest through transcriptional suppression of cell cycle regulators. (**A**) Heatmap showing differential gene expression profiles in MCF-7 cells following treatment with ARE (0.1, 0.3, and 0.5 mM). (**B**) Bubble plot representing the KEGG pathway enrichment analysis of significantly down-regulated genes. (**C**) Chord diagram illustrating the intricate interactions between the most enriched pathways and their associated key genes. (**D**) Flow cytometry analysis and quantification of cell cycle distribution in MCF-7 cells 12 h post-treatment, showing G1/S phase arrest. Data are expressed as mean ± SD (*n* = 3 biological replicates). *, *p* < 0.05, **, *p* < 0.01 vs. the untreated control (CTRL) group.

Building upon the phenotypic observations that ARE is a potent anti-proliferative agent, we performed a transcriptomic analysis (RNA-seq) on MCF-7 cells to delve deeper into its molecular mechanism of action. As shown in Figure 2A, MCF-7 cells treated with varying concentrations of ARE displayed a distinctly different gene expression profile compared to the control group, indicating that ARE induces widespread changes in gene transcription.

To identify the key signaling pathways driving this anti-proliferative effect, we conducted a KEGG functional enrichment analysis of the downregulated genes (Figure 2B). The results showed that among all enriched pathways, the “Cell cycle” pathway exhibited the most significant enrichment and contained the highest number of downregulated genes. Additionally, other pathways such as the “FoxO signaling pathway” and “Hippo signaling pathway,” which are closely related to cell cycle regulation, were also enriched. A chord diagram further visualized the interactions between these enriched pathways and their associated genes, highlighting that multiple critical cell cycle regulators, including *E2F1*, *E2F2*, *cyclin D1* (*CCND1*), and *cyclin E2* (*CCNE2*), were significantly downregulated after ARE treatment (Figure 2C).

To functionally validate this transcriptomic signature and directly quantify the effect of ARE on the cell cycle, we conducted flow cytometry analysis. As shown in Figure 2D, after 12 h of treatment, ARE induced a clear and significant G1/S phase arrest, evidenced by a dose-dependent accumulation of cells in the G1 phase and a corresponding reduction in the S and G2 phases.

### 3.3. Multi-Omics Convergence Identifies ATAD2 as the Direct Target of Arecoline

**Figure 3:**
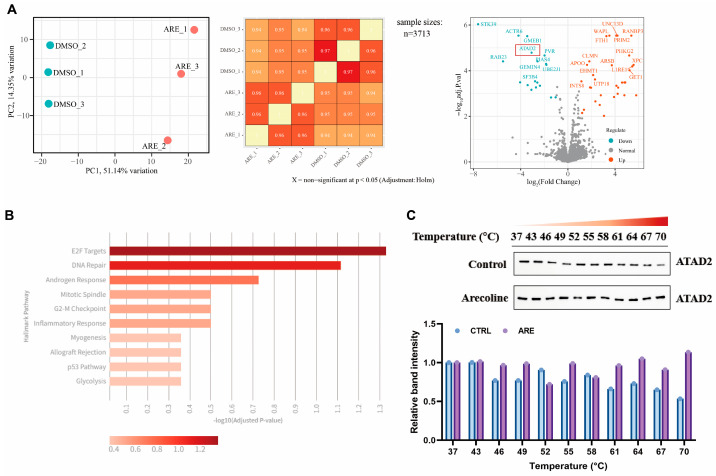
Identification and validation of ATAD2 as a target of ARE. (**A**) CETSA-MS analysis reveals a comprehensive set of potential protein targets for ARE. Principal component analysis (PCA) shows clear separation between treatment groups (ARE-treated samples, red; DMSO controls, cyan). (**B**) Top 10 enriched hallmark pathways results of ARE. (**C**) CETSA-WB validation confirms the direct interaction between ARE and ATAD2.

Given that the primary functional outcome of ARE treatment is the suppression of the E2F/Cell Cycle pathway, we next sought to identify potential direct protein targets that could mediate this cascade. We employed a high-throughput CETSA-MS screening approach, a powerful and unbiased method for identifying direct protein-ligand interactions within the native cellular environment based on ligand-induced thermal stabilization. The core principle of this technique is that ligand binding stabilizes the target protein against heat-induced denaturation. The CETSA-MS screening revealed 67 potential direct targets whose thermal stability was significantly altered upon ARE binding (Figure 3A).

To understand the collective function of these 67 biophysical targets, we performed a pathway enrichment analysis [32] on this list. Strikingly, this analysis revealed that the “E2F targets” pathway was identified as the most significantly enriched Hallmark pathway (Figure 3B). This finding provided a powerful convergence of evidence. Our transcriptomic analysis had already established that ARE’s primary functional outcome was the disruption of the E2F/Cell Cycle pathway. Now, this independent biophysical analysis indicated that the drug’s direct targets are, as a collective group, key regulators of that exact same pathway. This multi-omics convergence allowed us to confidently prioritize candidates, hypothesizing that the primary target would be a key upstream regulator of this pathway within our 67-hit list. This analysis led us directly to ATAD2. ATAD2 was identified as a robust, high-priority hit in our CETSA-MS screening. Critically, ATAD2 is a well-established transcriptional co-activator known to be essential for regulating E2F-driven gene expression, including many of the cyclins and E2F factors identified in our RNA-seq data. We acknowledge that the ATAD2 bromodomain is widely considered a challenging target for small-molecule inhibition, often due to the shallow and polar nature of its acetyl-lysine (KAc) binding pocket. However, our focus on ATAD2 was not based on a pre-selected hypothesis; rather, it was driven directly and unbiasedly by the convergence of our biophysical (CETSA-MS) and functional (transcriptomic) data. Thus, the selection of ATAD2 is supported by a strong, data-driven rationale: it represents the intersection of the top-ranked biophysical hits and the master regulators of the functionally suppressed pathway.

To further validate the direct interaction between ARE and ATAD2, we conducted a CETSA-WB analysis to assess the thermal stability of ATAD2 in a controlled temperature range from 37 °C to 70 °C. As shown in Figure 3C, ATAD2 in the DMSO-treated control group displayed a gradual loss of stability with increasing temperature. In sharp contrast, the ARE-treated group showed a notable enhancement in ATAD2’s thermal stability, particularly between 64 °C and 70 °C. Crucially, this observed thermal shift reflects ligand-induced stabilization of the existing protein pool, rather than an upregulation of protein expression levels. This thermal shift provides strong experimental evidence of a direct and stable binding interaction between ARE and the ATAD2 protein.

### 3.4. ARE Targets ATAD2 to Suppress the E2F/MYC-Driven Expression of G1/S Regulators

Our multi-omics approach identified ATAD2 as the primary target and E2F/Cell Cycle suppression as the primary functional outcome. We next sought to mechanistically link these two findings.

**Figure 4:**
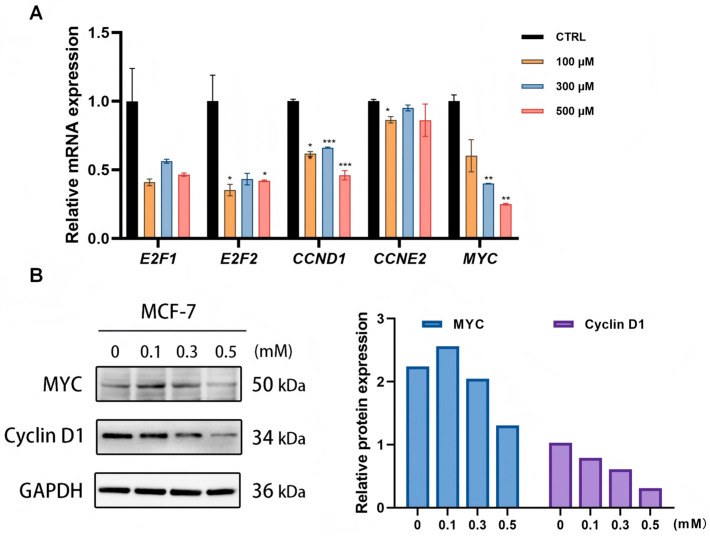
ARE inhibits G1/S phase progression in MCF-7 Cells. (**A**) Quantitative RT-PCR analysis of CCND1, CCNE2, and E2F1 expression levels after treatment with ARE at different concentrations (0.1, 0.3, and 0.5 mM). Data are presented as mean ± SD (*n* = 3). *, *p* < 0.05 vs. CTRL. **, *p* < 0.01 vs. CTRL. ***, *p* < 0.001 vs. CTRL. (**B**) Protein expression in MCF-7 cell lines treated by ARE.

Firstly, we confirmed the mRNA downregulation of these key ATAD2-regulated genes using quantitative RT-PCR. Consistent with our RNA-seq data, ARE treatment resulted in a significant dose-dependent downregulation of *CCND1*, *CCNE2*, and *E2F1* (Figure 4A).

More importantly, to determine if this mRNA suppression translates to a functional reduction at the protein level, we selected the pivotal G1/S regulators MYC and Cyclin D1 for Western Blotting (Figure 4B). MYC, a master transcription factor, and Cyclin D1, a key driver of CDK4/6 activity, are essential for cell cycle progression and frequently dysregulated in cancer [33,34]. The results showed that both MYC and Cyclin D1 expressions were significantly downregulated, consistent with the qPCR results, further indicating that ARE interferes with the normal regulation of the G1/S phase by inhibiting the expression of these critical proteins. Specifically, MYC, as a major transcription factor in the cell cycle, works in concert with Cyclin D1 to promote the transition from the G1 to the S phase. The downregulation of both proteins supports the mechanism by which ARE inhibits cell proliferation by affecting the expression of G1/S phase-related proteins. These findings provide strong experimental evidence for the potential of ARE as an anti-proliferative agent and suggest that ARE may exert its anti-cancer effects by targeting key regulators of the G1/S phase.

### 3.5. In Silico Evaluation of Arecoline Derivatives Reveals Improved Predicted Safety Profiles

To improve the binding affinity of ARE to ATAD2 and reduce potential toxicity, structural optimization was conducted. A customized version of PocketFlow [21] was developed to allow scaffold-guided molecular generation through atom-level constraints, enabling fixation of any user-defined core structure as an initial seed; The conserved ARE pharmacophore was fixed as the core scaffold. The customized PocketFlow model was applied to the ATAD2 KAc-binding pocket to generate a focused library of 10,000 ARE derivatives (Table S2). For each generated compound, docking against ATAD2 was performed, and candidates were retained only if they (i) preserved the ARE pharmacophore, (ii) achieved docking scores more favorable than both BOH and ARE, (iii) showed either extended occupation of the neighboring hydrophobic subpocket or additional polar interactions with pocket residues. These criteria formed the basis for selecting the six representative derivatives that were further analyzed in this work (Table 2).

These selected derivatives showed substantially improved predicted binding affinities, with AutoDock Vina scores ranging from −8.5 to −9.5 kcal/mol, compared to the parental ARE (−5.1 kcal/mol). A detailed analysis of their binding modes revealed the structural basis for this enhancement. For example, the pyrazole-based scaffolds, ARE_221 (−9.5 kcal/mol) and ARE_768 (−9.3 kcal/mol), introduce aromatic rings that extend into a neighboring hydrophobic subpocket formed by residues such as PHE1009 and LEU1011, establishing new van der Waals contacts. Similarly, ARE_632 (−9.0 kcal/mol) introduces a para-substituted aromatic ring that effectively fills this same hydrophobic subpocket near PHE1009 and TYR1021. Other derivatives, such as ARE_711 (−8.9 kcal/mol), demonstrate how modifications to the piperidine ring can improve shape complementarity. While all derivatives retained favorable drug-likeness (QED > 0.81), these specific structural extensions and new interactions provide a clear mechanistic explanation for their significantly improved binding scores over the parental compound.

**Figure 5:**
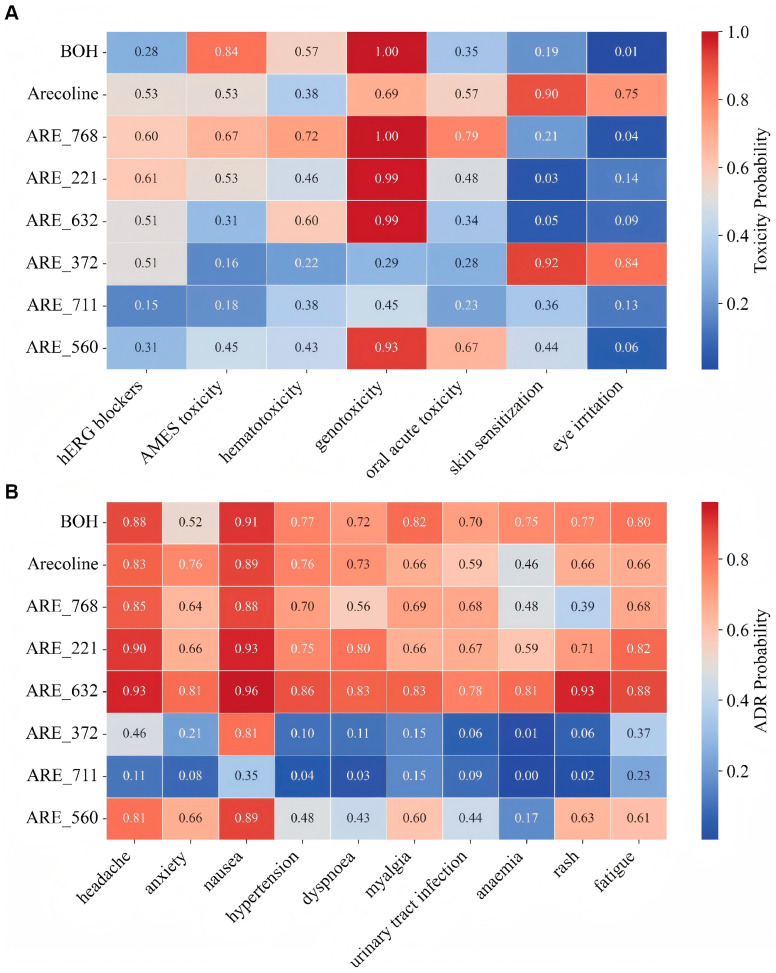
In silico prediction of safety and ADMET profiles for ARE and its derivatives. (**A**) Comparative analysis of predicted toxicity endpoints for the parental compound ARE and its derivatives using the ADMETLab 2.0 platform. (**B**) Predicted risk of adverse drug reactions (ADRs) associated with different organ systems, as determined by the DeepADR model. The heatmap color scale indicates the predicted probability of toxicity, ranging from low (blue) to high (red).

The six selected ARE derivatives were evaluated for toxicity and adverse drug reaction (ADR) risks using ADMETLab [26] and DeepADR [27] (Figure 5A,B). The parental compound ARE exhibited high predicted toxicity across multiple endpoints, including hERG blockers, AMES toxicity, genotoxicity, oral acute toxicity, skin sensitization, and eye irritation, as well as increased risks of adverse drug reactions involving multiple organ systems such as the nervous, cardiovascular, respiratory, musculoskeletal, and dermatologic systems. Within the nervous system category, the predicted ADRs included tremor, dizziness, agitation and salivary hypersecretion, which are consistent with the well-characterized muscarinic cholinergic responses associated with arecoline exposure [35]. In contrast, among the optimized derivatives, ARE_711 showed the most favorable safety profile, with substantially lower predicted toxicity and ADR (Table S3) probabilities across all assessed categories. Notably, ARE_711 did not exhibit these cholinergic-related nervous system ADRs in the prediction results, indicating that structural optimization effectively reduced this component of the risk profile. These results suggest that ARE_711 has a significantly improved risk profile compared to ARE, highlighting its potential as a safer structural analog.

### 3.6. Structural and Dynamic Analysis of Ligand–ATAD2 Interaction

The bromodomain of ATAD2 contains a conserved KAc-binding pocket that is indispensable for recognizing acetylated lysine residues on histones. This pocket is defined by a set of key amino acid residues (ASN1064, TYR1021, PHE1009, TYR1063, and ILE1074) [28], which together establish the hydrogen bonding, π stacking, and hydrophobic environment required for selective ligand engagement. ASN1064 plays a pivotal role in anchoring acetyl groups via hydrogen bonding, while TYR1021 and TYR1063 contribute to π-stacking interactions that stabilize planar ligands. PHE1009 and ILE1074 reinforce ligand binding through hydrophobic contacts, shaping the interior of the pocket.

**Figure 6:**
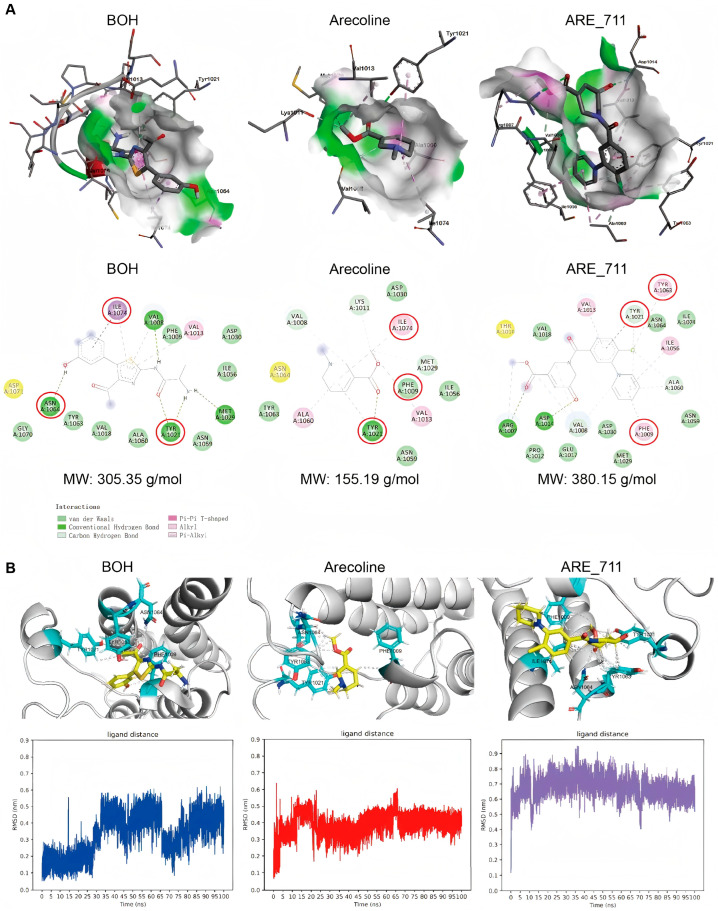
Structural and dynamic analysis of ligand binding to the ATAD2 bromodomain. (**A**) Comparative static docking poses of the reference ligand BOH, parental compound ARE, and derivative ARE_711 in the KAc-binding pocket. Note that the canonical hydrogen bond with ASN1064 is absent for ARE and ARE_711 in these static models. (**B**) Results of 100 ns Molecular Dynamics (MD) simulation analysis of ARE_711. A representative 3D snapshot of the final stabilized pose (**up**) and a plot of ligand Root Mean Square Deviation (RMSD) over time (**down**) are shown. The simulation demonstrates that, in contrast to the static model, ARE_711 achieves a stable, persistent hydrogen bond with ASN1064, providing a dynamic molecular basis for its improved predicted affinity.

Molecular docking analysis (Figure 6A) revealed that ARE exhibited favorable binding energy and engaged key hydrophobic residues, including ILE1074 and TYR1021, in a manner similar to the reference ligand BOH (a known ATAD2 positive control ligand). Hydrogen bonding with ASN1064, which is a central interaction in acetyl lysine recognition, was observed in the BOH–ATAD2 complex but absent in both ARE and ARE_711. Nevertheless, all three ligands consistently contacted TYR1021 and PHE1009, highlighting their structural role in ligand accommodation within the pocket.

To assess interaction persistence, molecular dynamics simulations were performed for 100 ns (Figure 6B). Trajectory analysis utilizing Root Mean Square Deviation (RMSD) confirmed the dynamic stability of the ligand–protein complex. In the final simulation frame, ARE_711 displayed the most extensive interaction profile with the KAc-binding pocket, maintaining hydrogen bonding with ASN1064 and stable hydrophobic or π–π interactions with TYR1021, TYR1063, PHE1009, and ILE1074. Although ARE_711 adopted a slightly greater average distance from the pocket centroid (~0.2 nm longer than BOH and ARE), its ability to preserve multiple simultaneous contacts suggests a robust engagement mode. These results indicate that ARE_711 achieves the most comprehensive occupancy of the KAc-binding pocket, effectively competing for the histone acetyl lysine binding surface and potentially disrupting native recognition events.

## 4. Discussion

In this study, we systematically investigated the molecular mechanism of ARE and its effects on cellular behavior. While ARE has been widely studied for its diverse biological activities, a direct molecular target remained to be identified. By employing a comprehensive multi-omics approach, we successfully identified and validated ATAD2 as a novel direct binding target of ARE. Our CETSA-MS screening pinpointed ATAD2 as a top candidate, and subsequent CETSA-WB analysis confirmed this direct interaction by showing that ARE binding significantly enhances the thermal stability of the ATAD2 protein. Molecular docking simulations further provided a structural basis for this finding, predicting that ARE occupies the acetyl-lysine (KAc) binding pocket of the ATAD2 bromodomain, primarily through key hydrophobic interactions with residues such as VAL1006, ILE1074, and VAL1013.

The direct binding of ARE to ATAD2 provides a crucial mechanistic link to the observed changes in cellular phenotype. We found that ARE treatment suppresses cellular viability and colony formation, a finding consistent with its known biological activities [36]. Our in-depth molecular analyses revealed that this functional effect is directly mediated by G1/S phase cell cycle arrest. Transcriptomic profiling showed that ARE induces a widespread downregulation of key cell cycle-related genes, including *E2F1*, *E2F2*, *MYC*, *CCND1*, and *CCNE2*. This finding is highly significant as these genes are well-established drivers of the G1/S transition, and their regulation is a central mechanism for controlling cell proliferation [33,37]. We provided compelling evidence for this model through both quantitative RT-PCR and Western blotting, which confirmed the downregulation of key proteins like MYC and Cyclin D1 at both the mRNA and protein levels, respectively. Collectively, these data support a model where ARE suppresses cell cycle progression, potentially by interfering with the regulatory network anchored by ATAD2. While further genetic perturbation studies, such as knockdown or overexpression, would be required to strictly establish exclusive causality, the convergence of direct binding data and downstream pathway suppression strongly implicates ATAD2 engagement as a primary driver of the observed phenotype.

The identification of ATAD2 as a direct target of ARE is particularly noteworthy given ATAD2’s function as an epigenetic reader. Bromodomains are protein domains that recognize and bind to acetylated lysine residues on histones, thereby “reading” epigenetic marks and linking them to transcriptional regulation [38]. As a conserved chromatin regulator, ATAD2’s function is to facilitate gene expression and chromatin dynamics [39]. Our findings suggest that ARE acts as a modulator of this epigenetic function, offering a new avenue for modulating the cell cycle.

Furthermore, our study provides a valuable translational perspective by demonstrating that the ARE scaffold can be rationally optimized for drug development. It is important to acknowledge that the Arecoline concentrations used in this study (0.1–0.5 mM), while consistent with previous mechanistic investigations in epithelial models, are higher than typical physiological plasma levels achievable through oral ingestion. We clarify that these concentrations were employed as scaffold-level doses for proof-of-concept target identification. The primary goal was to utilize Arecoline as a chemical probe to uncover the ATAD2-dependent mechanism, rather than to mimic clinical pharmacokinetics. The identification of ATAD2 as a direct target at these concentrations provides the structural basis for our subsequent in silico optimization. Based on our structural and mechanistic insights, we performed an optimization process that yielded derivatives with enhanced ATAD2 binding affinity and significantly reduced predicted toxicity. These findings highlight the potential for ARE to serve as a lead compound for the rational design of novel ATAD2 inhibitors. While further preclinical and in vivo studies are warranted to fully evaluate the efficacy and safety of these derivatives, this research provides a strong foundation for developing a new class of cell cycle modulators.

In addition to defining the ATAD2-centered mechanism, we also evaluated potential cross-target effects to place our findings in a broader pharmacological context. ARE is known to exert cholinergic stimulation and has been associated with central nervous system activity in long-term use. However, our CETSA-MS dataset did not identify stabilization of MAO-A, dopamine receptors, or other canonical CNS-related targets. This observation is consistent with published literature indicating that many acute biological responses to ARE arise through peripheral muscarinic pathways with no clear evidence supporting involvement of dopaminergic or MAO-A–mediated mechanisms [35,40]. Nonetheless, given ARE’s reported CNS activity in other settings, a more comprehensive off-target profiling strategy, including expanded proteomic screening across neural cell types or receptor-focused assays, will be important for future work. These additional investigations will help clarify how ATAD2-targeted activity fits within the broader pharmacology of ARE and ensure that optimized analogs maintain favorable specificity profiles.

## 5. Conclusions

This study establishes ATAD2 as the primary molecular target mediating ARE’s cellular effects. We demonstrate that ARE binds directly to ATAD2, leading to the coordinated suppression of key G1/S phase drivers, including CCND1, CCNE2, and E2F1. Our structural optimization efforts successfully generated derivatives with superior binding affinity for ATAD2 and a significantly improved safety profile. These findings not only provide a clear mechanistic basis for ARE’s biological activities but also underscore the potential of its scaffold for the rational design of novel ATAD2 inhibitors with enhanced properties for cell cycle modulation.

## Figures and Tables

**Table 1:** Primers used for qPCR assay.

Name	Sequence	
*E2F1*	Forward Primer (5′→ 3′)	ACGCTATGAGACCTCACTGAA
	Reverse Primer (5′→ 3′)	TCCTGGGTCAACCCCTCAAG
*E2F2*	Forward Primer (5′→ 3′)	CGTCCCTGAGTTCCCAACC
	Reverse Primer (5′→ 3′)	GCGAAGTGTCATACCGAGTCTT
*CCNE2*	Forward Primer (5′→ 3′)	TCAAGACGAAGTAGCCGTTTAC
	Reverse Primer (5′→ 3′)	TGACATCCTGGGTAGTTTTCCT
*MYC*	Forward Primer (5′→ 3′)	GGCTCCTGGCAAAAGGTCA
	Reverse Primer (5′→ 3′)	CTGCGTAGTTGTGCTGATGT
*CCND1*	Forward Primer (5′→ 3′)	GCTGCGAAGTGGAAACCATC
	Reverse Primer (5′→ 3′)	CCTCCTTCTGCACACATTTGAA
*GAPDH*	Forward Primer (5′→ 3′)	TGTGGGCATCAATGGATTTGG
	Reverse Primer (5′→ 3′)	ACACCATGTATTCCGGGTCAAT

**Table 2:** ARE-derived ATAD2 inhibitors for structural optimization.

Name	Scaffold Type	Structure	Vina Score
BOH	Control	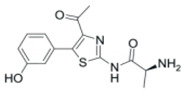	−7.1
ARE	Control (Piperidine)	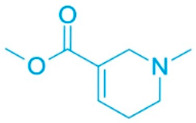	−5.1
Piperidine Derivatives			
ARE_372	Piperidine	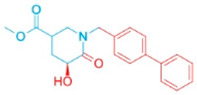	−8.5
ARE_711	Piperidine	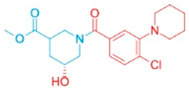	−8.9
ARE_560	Piperidine	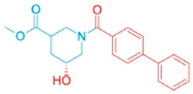	−8.5
Pyrazole Scaffolds			
ARE_768	Pyrazole	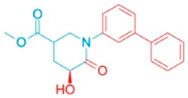	−9.3
ARE_221	Pyrazole	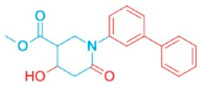	−9.5
ARE_632	Pyrazole	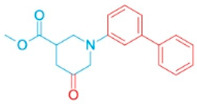	−9.0

## Data Availability

The original contributions presented in this study are included in the article/Supplementary Material. Further inquiries can be directed to the corresponding authors.

## Supplementary Materials

The following supporting information can be downloaded at: https://www.mdpi.com/article/10.3390/pharmaceutics18030324/s1, Table S1: Chromatographic Gradient and SWATH-MS Acquisition Windows; Table S2: Library of arecoline-derived compounds generated by the customized PocketFlow model targeting ATAD2; Table S3: Predicted toxicity and ADR risks of the selected ARE derivatives.

## Abbreviations

The following abbreviations are used in this manuscript:

ATAD2	ATPase family AAA domain-containing protein 2
ARE	Arecoline
CETSA-MS	Cellular Thermal Shift Assay–Mass Spectrometry
CETSA-WB	Cellular Thermal Shift Assay–Western Blot
DEGs	Differentially Expressed Genes
E2F1	E2F transcription factor 1
E2F2	E2F transcription factor 2
CCND1	Cyclin D1
CCNE2	Cyclin E2
MYC	MYC proto-oncogene, bHLH transcription factor
SA	Synthetic Accessibility
QED	Quantitative Estimate of Drug-likeness
log*P*	Partition Coefficient

## References

[1] Ciró M., Prosperini E., Quarto M., Grazini U., Walfridsson J., McBlane F., Nucifero P., Pacchiana G., Capra M., Christensen J. (2009). ATAD2 is a novel cofactor for MYC, overexpressed and amplified in aggressive tumors. Cancer Res..

[2] Boussouar F., Jamshidikia M., Morozumi Y., Rousseaux S., Khochbin S. (2013). Malignant genome reprogramming by ATAD2. Biochim. Biophys. Acta.

[3] Kalashnikova E.V., Revenko A.S., Gemo A.T., Andrews N.P., Tepper C.G., Zou J.X., Cardiff R.D., Borowsky A.D., Chen H.W. (2010). ANCCA/ATAD2 overexpression identifies breast cancer patients with poor prognosis, acting to drive proliferation and survival of triple-negative cells through control of B-Myb and EZH2. Cancer Res..

[4] Fu J., Zhang J., Chen X., Liu Z., Yang X., He Z., Hao Y., Liu B., Yao D. (2023). ATPase family AAA domain-containing protein 2 (ATAD2): From an epigenetic modulator to cancer therapeutic target. Theranostics.

[5] Yuan L., Li S., Zhu Y., Yang L., Zhang X., Qu Y., Wang Z., Duan J., Zhong J., Tian Y. (2025). ATAD2 is a potential immunotherapy target for patients with small cell lung cancer harboring HLA-A *0201. EBioMedicine.

[6] Zhou Y., Hussain M., Kuang G., Zhang J., Tu Y. (2018). Mechanistic insights into peptide and ligand binding of the ATAD2-bromodomain via atomistic simulations disclosing a role of induced fit and conformational selection. Phys. Chem. Chem. Phys..

[7] Sanchez-Vega F., Mina M., Armenia J., Chatila W.K., Luna A., La K.C., Dimitriadoy S., Liu D.L., Kantheti H.S., Saghafinia S. (2018). Oncogenic Signaling Pathways in the Cancer Genome Atlas. Cell.

[8] Chang M.C., Ho Y.S., Lee P.H., Chan C.P., Lee J.J., Hahn L.J., Wang Y.J., Jeng J.H. (2001). Areca nut extract and arecoline induced the cell cycle arrest but not apoptosis of cultured oral KB epithelial cells: Association of glutathione, reactive oxygen species and mitochondrial membrane potential. Carcinogenesis.

[9] Tsai Y.S., Lee K.W., Huang J.L., Liu Y.S., Juo S.H., Kuo W.R., Chang J.G., Lin C.S., Jong Y.J. (2008). Arecoline, a major alkaloid of areca nut, inhibits p53, represses DNA repair, and triggers DNA damage response in human epithelial cells. Toxicology.

[10] Huang L.W., Hsieh B.S., Cheng H.L., Hu Y.C., Chang W.T., Chang K.L. (2012). Arecoline decreases interleukin-6 production and induces apoptosis and cell cycle arrest in human basal cell carcinoma cells. Toxicol. Appl. Pharmacol..

[11] Jeng J.H., Chang M.C., Hahn L.J. (2001). Role of areca nut in betel quid-associated chemical carcinogenesis: Current awareness and future perspectives. Oral Oncol..

[12] Zhou Z.S., Li M., Gao F., Peng J.Y., Xiao H.B., Dai L.X., Lin S.R., Zhang R., Jin L.Y. (2013). Arecoline suppresses HaCaT cell proliferation through cell cycle regulatory molecules. Oncol. Rep..

[13] Feng S.D., Wu D., Yang S.S., He J.Q., Zhang K.F., Ling H.Y. (2016). Effect of arecoline on proliferation and apoptosis of MCF-7 human breast cancer cells. Zhongguo Ying Yong Sheng Li Xue Za Zhi.

[14] Zhu F.Y., Chen M.X., Ye N.H., Qiao W.M., Gao B., Law W.K., Tian Y., Zhang D., Zhang D., Liu T.Y. (2018). Comparative performance of the BGISEQ-500 and Illumina HiSeq4000 sequencing platforms for transcriptome analysis in plants. Plant Methods.

[15] Zhou Y., Zhou B., Pache L., Chang M., Khodabakhshi A.H., Tanaseichuk O., Benner C., Chanda S.K. (2019). Metascape provides a biologist-oriented resource for the analysis of systems-level datasets. Nat. Commun..

[16] Lyu J., Ruan C., Zhang X., Wang Y., Li K., Ye M. (2020). Microparticle-Assisted Precipitation Screening Method for Robust Drug Target Identification. Anal. Chem..

[17] Demichev V., Messner C.B., Vernardis S.I., Lilley K.S., Ralser M. (2020). DIA-NN: Neural networks and interference correction enable deep proteome coverage in high throughput. Nat. Methods.

[18] Rosenberger G., Koh C.C., Guo T., Röst H.L., Kouvonen P., Collins B.C., Heusel M., Liu Y., Caron E., Vichalkovski A. (2014). A repository of assays to quantify 10,000 human proteins by SWATH-MS. Sci. Data.

[19] Ritchie M.E., Phipson B., Wu D., Hu Y., Law C.W., Shi W., Smyth G.K. (2015). limma powers differential expression analyses for RNA-sequencing and microarray studies. Nucleic Acids Res..

[20] Kurien B.T., Scofield R.H. (2006). Western blotting. Methods.

[21] Jiang Y., Zhang G., You J., Zhang H., Yao R., Xie H., Zhang L., Xia Z., Dai M., Wu Y. (2024). PocketFlow is a data-and-knowledge-driven structure-based molecular generative model. Nat. Mach. Intell..

[22] Ertl P., Schuffenhauer A. (2009). Estimation of synthetic accessibility score of drug-like molecules based on molecular complexity and fragment contributions. J. Cheminform..

[23] Bickerton G.R., Paolini G.V., Besnard J., Muresan S., Hopkins A.L. (2012). Quantifying the chemical beauty of drugs. Nat. Chem..

[24] Lipinski C.A., Lombardo F., Dominy B.W., Feeney P.J. (1997). Experimental and computational approaches to estimate solubility and permeability in drug discovery and development settings. Adv. Drug Deliv. Rev..

[25] Trott O., Olson A.J. (2010). AutoDock Vina: Improving the speed and accuracy of docking with a new scoring function, efficient optimization, and multithreading. J. Comput. Chem..

[26] Fu L., Shi S., Yi J., Wang N., He Y., Wu Z., Peng J., Deng Y., Wang W., Wu C. (2024). ADMETlab 3.0: An updated comprehensive online ADMET prediction platform enhanced with broader coverage, improved performance, API functionality and decision support. Nucleic Acids Res..

[27] Wan J., Jia C., Dong D., Chen Y., Lin Y.-C.-D., He Y., Huang H.-Y., Huang H.-D. (2026). DeepADR: Multimodal prediction of adverse drug reaction frequency by integrating early-stage drug discovery information via Kolmogorov–Arnold networks. Brief. Bioinform..

[28] Dolbois A., Batiste L., Wiedmer L., Dong J., Brütsch M., Huang D., Deerain N.M., Spiliotopoulos D., Cheng-Sánchez I., Laul E. (2020). Hitting a Moving Target: Simulation and Crystallography Study of ATAD2 Bromodomain Blockers. ACS Med. Chem. Lett..

[29] Liu Y., Yang X., Gan J., Chen S., Xiao Z.X., Cao Y. (2022). CB-Dock2: Improved protein-ligand blind docking by integrating cavity detection, docking and homologous template fitting. Nucleic Acids Res..

[30] Van Der Spoel D., Lindahl E., Hess B., Groenhof G., Mark A.E., Berendsen H.J. (2005). GROMACS: Fast, flexible, and free. J. Comput. Chem..

[31] Park H., Paganetti H., Schuemann J., Jia X., Min C.H. (2021). Monte Carlo methods for device simulations in radiation therapy. Phys. Med. Biol..

[32] Liberzon A., Birger C., Thorvaldsdóttir H., Ghandi M., Mesirov J.P., Tamayo P. (2015). The Molecular Signatures Database (MSigDB) hallmark gene set collection. Cell Syst..

[33] Dang C.V. (2012). MYC on the path to cancer. Cell.

[34] Musgrove E.A., Caldon C.E., Barraclough J., Stone A., Sutherland R.L. (2011). Cyclin D as a therapeutic target in cancer. Nat. Rev. Cancer.

[35] Papke R.L., Horenstein N.A., Stokes C. (2015). Nicotinic Activity of Arecoline, the Psychoactive Element of “Betel Nuts”, Suggests a Basis for Habitual Use and Anti-Inflammatory Activity. PLoS ONE.

[36] Badawy E., Abouelsaoud K., Ragab A., Kabbash A. (2023). Arecoline Biological Activity and Biotransformation: A review. J. Adv. Med. Pharm. Res..

[37] Rouaud F., Hamouda-Tekaya N., Cerezo M., Abbe P., Zangari J., Hofman V., Ohanna M., Mograbi B., El-Hachem N., Benfodda Z. (2018). E2F1 inhibition mediates cell death of metastatic melanoma. Cell Death Dis..

[38] Mujtaba S., Zeng L., Zhou M.M. (2007). Structure and acetyl-lysine recognition of the bromodomain. Oncogene.

[39] Koo S.J., Fernández-Montalván A.E., Badock V., Ott C.J., Holton S.J., von Ahsen O., Toedling J., Vittori S., Bradner J.E., Gorjánácz M. (2016). ATAD2 is an epigenetic reader of newly synthesized histone marks during DNA replication. Oncotarget.

[40] Liu H., Zheng H., Zhang J., Chen F., Hu X., Wang X. (2024). Review of the toxic effects and health functions of arecoline on multiple organ systems. Food Innov. Adv..

